# Towards the use of a smartphone imaging-based tool for point-of-care detection of asymptomatic low-density malaria parasitaemia

**DOI:** 10.1186/s12936-021-03894-w

**Published:** 2021-09-25

**Authors:** Ashlee J. Colbert, Katrina Co, Giselle Lima-Cooper, Dong Hoon Lee, Katherine N. Clayton, Steven T. Wereley, Chandy C. John, Jacqueline C. Linnes, Tamara L. Kinzer-Ursem

**Affiliations:** 1 Weldon School of Biomedical Engineering, Purdue University, West Lafayette, IN 47907 USA; 2 Indiana University School of Medicine, Indiana University, Indianapolis, IN 46202 USA; 3 School of Mechanical Engineering, Purdue University, West Lafayette, IN 47907 USA; 4 OmniVis Inc., South San Francisco, CA 94080 USA

**Keywords:** Malaria, Particle-diffusometry, Nucleic-acid based tests, Smartphone-detection, LAMP-assay

## Abstract

**Background:**

Globally, there are over 200 million cases of malaria annually and over 400,000 deaths. Early and accurate detection of low-density parasitaemia and asymptomatic individuals is key to achieving the World Health Organization (WHO) 2030 sustainable development goals of reducing malaria-related deaths by 90% and eradication in 35 countries. Current rapid diagnostic tests are neither sensitive nor specific enough to detect the low parasite concentrations in the blood of asymptomatic individuals.

**Methods:**

Here, an imaging-based sensing technique, particle diffusometry (PD), is combined with loop mediated isothermal amplification (LAMP) on a smartphone-enabled device to detect low levels of parasitaemia often associated with asymptomatic malaria. After amplification, PD quantifies the Brownian motion of fluorescent nanoparticles in the solution during a 30 s video taken on the phone. The resulting diffusion coefficient is used to detect the presence of *Plasmodium* DNA amplicons. The coefficients of known negative samples are compared to positive samples using a one-way ANOVA post-hoc Dunnett’s test for confirmation of amplification.

**Results:**

As few as 3 parasite/µL of blood was detectable in 45 min without DNA extraction. *Plasmodium falciparum* parasites were detected from asymptomatic individuals’ whole blood samples with 89% sensitivity and 100% specificity when compared to quantitative polymerase chain reaction (qPCR).

**Conclusions:**

PD-LAMP is of value for the detection of low density parasitaemia especially in areas where trained personnel may be scarce. The demonstration of this smartphone biosensor paired with the sensitivity of LAMP provides a proof of concept to achieve widespread asymptomatic malaria testing at the point of care.

**Supplementary Information:**

The online version contains supplementary material available at 10.1186/s12936-021-03894-w.

## Background

Malaria is a crucial public health concern in resource-constrained countries. In 2018, there were 228 million cases of malaria and 405,000 malaria related deaths worldwide [1]. Despite the World Health Organization’s (WHO) strategic goal to eradicate malaria in 10 countries and reduce global incidence by 40% by 2020, malaria cases have increased in the past several years [2, 3]. Countries throughout sub-Saharan Africa carry the greatest percentage of malaria cases (92%), followed by countries in Southeast Asia (5%) [2]. One contributing factor to the disproportionate number of malaria cases in sub-Saharan Africa is delayed or inaccurate results along with a lack of access to malarial diagnostic tools that are practical for field use [4]. There is a need for portable, prompt, and easy-to-use diagnostic tools to decrease mortality from such a curable and preventable disease [5].

Malaria is caused by the protozoan parasite *Plasmodium* with *Plasmodium falciparum* being the deadliest in Africa [2, 4]. When *P. falciparum* malaria is left untreated it can become fatal and accounts for 99% of malaria deaths [2]. Malaria symptoms are nonspecific and often mimic symptoms of common viral and bacterial illnesses [6]. However, a large proportion of malaria cases are asymptomatic in endemic countries [7–9]. Asymptomatic carriers of *P. falciparum* malaria are largely responsible for persistent transmission by maintaining the parasite life cycle [8, 10, 11]. Malaria management strategies are needed to control and monitor infections in asymptomatic carriers and are a key step towards elimination [10].

Rapid and sensitive asymptomatic malaria detection would provide real-time disease surveillance for disease outbreak identification and prevent further transmission. However, current on-site malaria detection approaches often rely on diagnostics that are not sensitive enough for asymptomatic malaria cases [12, 13]. The standard method for diagnosing malaria is through microscopic examination of blood smears. Microscopy achieves a sensitivity of 50–100 parasites/µL [12, 14–17]. Major drawbacks of microscopy include the need for extensive technical training for skilled personnel and the lack of quality control that is introduced in the diagnostic interpretation [12]. An alternative approach, polymerase chain reaction (PCR), is by far the most sensitive malaria diagnostic on the market with a limit of detection (LOD) of 5 parasites/µL, but requires expensive equipment and reagents not found in local clinical facilities [18, 19]. Additionally, rapid diagnostic tests (RDTs) have been developed as a simple point-of-care alternative for malaria diagnosis, which require little technical training and no laboratory infrastructure [20]. However, common RDTs are currently not sensitive enough to accurately detect below 100 parasites/µL of blood, a concentration too high to identify malaria in some asymptomatic individuals [21–24].

Isothermal amplification methods eliminate the thermal cycling that is needed for highly accurate methods such as PCR and have the ability to robustly amplify nucleic acids in complex matrices while maintaining sensitivity and specificity [25, 26]. This simplifies the process and decreases the time from sample to answer [25, 26]. The use of one such isothermal technique, loop mediated isothermal amplification (LAMP) is an attractive nucleic acid amplification technique for field use due to its simplicity and robustness in complex matrices in comparison to other isothermal methods [27]. LAMP-based assays have also been deemed appropriate for detection of low-level parasitaemia with the commercialized Loopamp™ malaria Pan/Pf kit by having an excellent limit of detection of 2 parasites/µL [27, 28]. However, DNA still needs to be extracted from the organism to use the Eiken kit. LAMP detection from blood without sample purification has been done previously with 1000 fold dilution of the blood samples and blood spots using chemical lysis through commercialized Loompamp kits [23, 29]. However these methods are commonly analysed based on fluorescence detection adding to testing complexity but can be expounded upon to increase its compatibility for field use.

LAMP is often monitored by turbidity, fluorescence, and electrochemical methods [14, 30, 31]. To accurately measure these signals, many research groups have begun to rely on the use of smartphones as sensing instruments. Smartphones are the next leading technology in the medical field as they are an attractive alternative to expensive medical equipment, oftentimes contain a camera, GPS capabilities, and vibrational sensors that can be manipulated for implementation of advanced diagnostics [32]. Smartphone-enabled LAMP-based diagnostics for detection of *Plasmodium* has been performed previously and shows promise for assisted microfluidic lab-on-a-chip devices [20, 33]. With developments on previous works, a feasible point-of-care diagnostic can be developed to combat the challenges present in parasite detection including laborious DNA extraction, purification and low-level malaria parasite detection in one device.

**Fig. 1:**
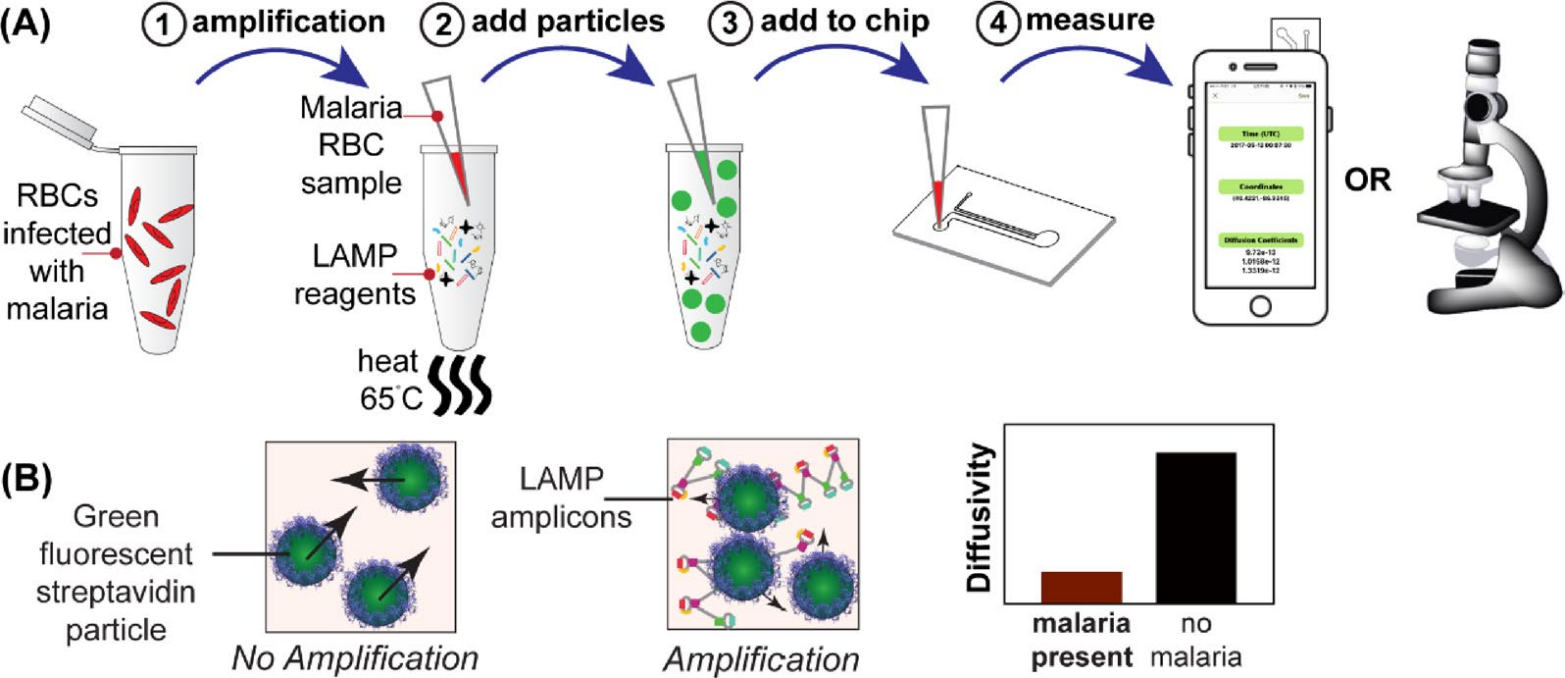
Illustration of PD-LAMP set-up. **A** LAMP was performed with whole blood samples added directly to the assay reagents. **A1** The red blood cells lysed upon heating, releasing the malaria DNA and initiating the LAMP reaction. **A2** After LAMP is completed, the amplicons are combined with fluorescent particles and **A3** the mixture was then added to a microfluidic chip. **A4** Imaging of the fluorescent beads took place using an epifluorescent microscope or the smartphone device (developed by Moehling et al.) [36]. **B** The smartphone images the fluorescent particles undergoing Brownian motion for 30 s. The particles will exhibit faster Brownian motion in the absence of DNA amplicons. In the presence of malaria DNA, the particle motion will be hindered. The diffusion coefficient value, a numerical measure of Brownian motion, is lower when malaria DNA is amplified than if no malaria DNA is present

An alternative highly sensitive detection method applying the optical sensing technique is particle diffusometry paired with LAMP (PD-LAMP). PD-LAMP has previously been demonstrated by Clayton et al. for the environmental detection of *Vibrio cholerae* [34]. The presence of a pathogen is detected by measuring the Brownian motion of particles in solution after LAMP. The LAMP assay, with biotinylated primers incorporated, produces approximately 10^9^ copies of target DNA amplicons. These amplicons consist of stem-loops with varying lengths; increasing the viscosity of the sample drastically (Fig. 1A) [16, 35]. These LAMP amplicons are then combined with 400 nm streptavidin-coated fluorescent particles and placed into a microfluidic chip (Fig. 1A). The fluorescent particles bind the biotinylated DNA primer that is hybridized into the DNA amplicons resulting in an increase in the hydrodynamic radius of the particles, further slowing their Brownian motion. Movement of the fluorescent particles is captured in a series of images via the smart-phone camera. Correlation-based algorithms of the images are used to calculate the diffusion coefficient of the particles (Fig. 1B). Combined, the particle size change and increased fluid viscosity yields a significant difference between the diffusion coefficients of particles in positive *versus* negative samples [34]. Diffusion coefficients are low in the presence of the targeted pathogen and in the absence of pathogen nanoparticles will exhibit higher diffusivity. Clayton et al. used PD-LAMP to sensitively and specifically detect 10 V*. cholerae* cells in a 25 µL reaction in pond water within 35 min [34]. However, this technique was performed using a laboratory epifluorescence microscope, a method that is not easily accessible at the point of care. More recently, Moehling et al*.* expanded upon the PD-LAMP method, achieving the same limit of detection of *V. cholerae* cells in pond water using a newly developed smartphone-enabled detection platform [36]. Their newly developed portable device miniaturizes a fluorescent microscope and takes advantage of the smartphone camera and computational power needed to perform PD-LAMP [36].

Here, low concentrations of *Plasmodium* parasites in unprocessed blood is detected using PD-LAMP. A previously published primer set targeting the 18 s rRNA gene [37] that is specific to *P. falciparum* is used alone and in combination with a novel 6-primer LAMP assay to amplify pan-*Plasmodium* targeting the 28 s rRNA gene, which is conserved across all malaria species. The resulting smartphone-enabled PD-LAMP assay is a rapid, specific, and robust method for the detection of asymptomatic malaria in a portable detection platform.

## Methods

### Loop mediated isothermal amplification (LAMP)

Purified genomic DNA from *P. falciparum* 3D7 (UK repository) was stored at 66 ng/µL (3 × 10^6^ copies/µL) at − 20 °C. The stock was diluted tenfold (3 × 10^0^–10^4^ copies/reaction) in molecular biology water (Invitrogen, Carlsbad, CA) for experiments. The 28 s rRNA LAMP primers were designed using primer explorer to target the *Plasmodium* 28 s rRNA, a gene conserved across all *Plasmodium*. 18 s rRNA, another conserved gene, primers used in this study were adopted from Lau et al. [37]. Primer sequences can be found in Additional file 1: Table S1 and S2.

For all amplification experiments a master mix of 22.5 µL (master mix recipe found in Additional file 1: Table S3) and 2.5 µL of template or NTC were added just prior to heating. The templates were genomic DNA or infected blood; negative template controls (NTCs) were of molecular biology water or uninfected blood, respectively. The reactions were heated at 65 °C for 45 min for all reactions without blood and all clinical samples with the 18 s primer set, 60 min for specificity reactions with blood, or 75 min for sensitivity reactions with blood using an Applied Biosystems 7500 Real time PCR System (Foster City, CA). Samples were stored at 4 °C before PD analysis. Samples are prepared in a separate location from amplification, gel electrophoresis and PD to limit contamination. Amplified samples never encountered the space used to prepare LAMP reactions.

LAMP products were visualized using an ethidium bromide stained 2% agarose gel at 100 V for 50 min. The gel was imaged using an ultraviolet light gel system (c400, Azure Biosystems, Dublin, CA). Gel images were collected with an exposure time of 15 s using the Azure Series software at settings of UV302. Gel images were transferred from the Azure as.tiff files and have not been cropped or edited in this manuscript.

### PD-LAMP imaging platform

The optics for the smartphone-based PD platform are housed in a 3D printed case built for use with an iPhone 6 (Additional file 1: Figure S9). The 3D printed housing was designed using SolidWorks. The case was designed to incorporate a power source, a secure slot to image the microfluidic chip, and a 445 nm (blue) laser (Laserland, Wuhan,CN), an R12 straw film filter blocking excess blue light (Rosco, Stamford, CT), and a 0.5 mm N-BK7 ball lens from Edmonds Optics mounted in a custom machined metal plate in front of the iPhone 6 camera (see [36] for images).

### PD-LAMP particle preparation and imaging

For PD measurements, 400 nm streptavidin coated Dragon Green polystyrene beads (Bangs Laboratories, Fishers, IN) were added to the LAMP samples at a final concentration of 6 × 10^9^ particles/mL. After 10 s of microcentrifugation 3 µL of the LAMP and bead sample was placed into the microfluidic chip imaging chamber. Each sample was imaged for 30 s in the smartphone-enabled platform twice and analyzed using an in-house algorithm [36]. The diffusion coefficients were exported for statistical analysis.

For the microscope images, the samples (N = 3) were imaged for 1000 frames using an inverted fluorescence microscope (Carl Zeiss Microscopy, Thornwood, NY) [34]. A 40× magnification objective lens was used with the ZEN software and Zeiss camera at 13.5 frames per second. A representative image is shown in Additional file 1: Figure S7. Each sample video from the microscope was analysed using an in-house MATLAB code to obtain diffusion coefficients (Additional file 2: Video S1 and Additional file 3: Video S2).

### Chip preparation

The microfluidic chip (Additional file 1: Figure S8) for phone measurements was manufactured by pressure and heat with a heat press (Carver Inc. Wabash, IN). The chip consisted of two 188 µm thick cyclic olefin polymer (COP) sheets (Zeon, Tokyo, Japan) that were adhered together at 1.2 metric tons at 120 °C for 2 min and then rotating the COP sheets 180° and pressing for another 1 min. Double-sided pressure sensitive adhesive (PSA) (120 µm thickness iCraft Super Tape) had a 1/8″ (3 mm) through-hole fabricated with a hole punch. The PSA was placed on one side of the pressed 188 µm sheets. The PSA acted as the fluid sample well. A 60 µm COP sheet was placed on top of the PSA after adding the sample to prevent evaporation.

For the samples analysed via microscope, 6 mm punches were used on PSA to act as a sample well. The PSA was placed onto a cover glass slide (Thickness No. 1, Thermo Scientific, Erie, NY, USA) 3 µL sample was placed into the wells and sealed with a second cover glass slide to limit evaporation [34].

### Theory of particle diffusometry

PD-LAMP is a correlation-based fluid visualization technique that utilizes imaging of Brownian motion of particles post-DNA amplification [38–40]. Diffusion coefficients are calculated by correlating sequential particle images and using autocorrelation and cross-correlation of these images to measure particle pixel displacement. The greater particle displacement between images creates a broader cross-correlation peak width, $s_{c}$. The autocorrelation coefficient $s_{a}$ is determined by correlating an image captured at time $t$ with itself. Through the use of these correlation coefficients the diffusion coefficient can be calculated by an equation derived by Olsen and Adrian [41]:

1 $$ D = \frac{{s_{c}^{2} - s_{a}^{2} }}{{16 M^{2} \Delta t}} $$

where M is the magnification of the microscope objective. D is the diffusion coefficient where its theoretical value is calculated using the Stokes–Einstein equation [42, 43].

2 $$ D = \frac{kT}{{6\pi \eta a}} $$

Here k is the Boltzmann constant, T is the absolute temperature, $\eta$ is the viscosity and a is the hydrodynamic radius of the imaged fluorescent particles. The Eqs. (1) and Eq. (2) and its use in PD-LAMP have been previously described [34, 36, 39].

### Patient/study participant samples and malarial DNA

Genomic *P. falciparum* 3D7A DNA was obtained from the European Malaria Reagent Repository. NIAID, NIH *P. falciparum* strain 3D7, MRA-102, contributed by Daniel J. Carucci, and NIAID, NIH *Plasmodium vivax* strain Chesson, MRA-383, contributed by W. E. Collins, were obtained through BEI Resources. Human blood (Innovative Research Novi, MI) was used to dilute infected blood samples.

Cerebral malaria (CM), severe malarial anemia (SMA), and community control (CC) blood samples were collected as part of a study conducted at Mulago National Referral Hospital in Kampala, Uganda from 2008 to 2013 as previously described [44]. Children 18 months to 12 years of age were enrolled into the CM group if they had coma (Blantyre Coma Score ≤ 2), *P. falciparum* on blood smear, and no other known cause of coma (e.g., meningitis, a prolonged postictal state, or hypoglycemia-associated coma reversed by a glucose infusion) or into the SMA group if they had *P. falciparum* on blood smear and serum Hgb ≤ 5 mg/dL. CC were healthy children in the same age group and from the same neighbourhood, extended household, or nearby neighbourhood as a child with CM. Whole blood was also obtained from a Kenyan individual with uncomplicated malaria (UM) and from North American individuals without malaria (IRB Protocol 1601403732). DNA was extracted from whole blood samples using the QIAamp DNA Blood Mini Kits (Qiagen, Hilden, Germany) for nested PCR (nPCR) and quantitative PCR (qPCR) testing.

### Blinded study

*Plasmodium falciparum* positive or negative patient samples (2.5 µL) were placed into a PCR strip tube labelled 1–7. The master mix was prepared, and the samples added. Samples were heated to 65 °C for 45 min for the 18 s primer set and 90 min for the 28 s primer set. The researchers performing LAMP-amplification and PD experiments were blinded to the parasitaemia sample concentration. After PD-LAMP was performed, the diffusion coefficients were then matched with the initial concentrations to obtain unbiased measurements.

### Nested polymerase chain reaction

nPCR was used to first amplify the genus specific 18 s ribosomal RNA common to all *Plasmodium* species using the rPLU1 and rPLU5 primers as described in the Snounou protocol [45] and the cycling method as described in the Bharti protocol [46]. Then, the product of this first reaction was used as the DNA template for the second, *P. falciparum* species-specific amplification using the rFAL1 and rFAL2 primers as described in the Snounou protocol with the same cycling conditions as the first amplification.

### Quantitative polymerase chain reaction

qPCR targeting the multi-copy nuclear *var*ATS gene was performed on the study participant samples that also underwent PD-LAMP. 20 µL reactions were run on the Applied Biosystems™ QuantStudio™ 6 Flex Real-Time PCR System (Foster, CA) using primers and protocol previously described [47] but modified for PowerUp™ SYBR™ Green Master Mix (Applied Biosystems, Foster, CA) (Additional file 1: Table S4). Parasite density was quantified by comparison to a standard curve of 3D7 parasite cultures. After two rounds of synchronization (5% sorbitol), parasite concentration was determined using the parasitemia calculation described below and the RBC concentration via hemocytometer. A tenfold serial dilution of *P. falciparum* parasite culture in RPMI diluted in malaria-negative O + blood was produced and the DNA isolated using the QIAamp DNA Blood Mini Kits (Qiagen, Hilden, Germany) to create the standards (1 × 10^5^–1 × 10^–1^ parasites/µL). All standards, controls, and samples were quantified in duplicate and averaged.

### Parasitaemia and parasite density calculations

Parasitaemia of the NIAID, NIH *P. falciparum* strain 3D7, MRA-102, and NIH *P. vivax* strain Chesson, MRA-383, containing live parasites, were verified by microscopy. Microscopy was performed on a sample of 1.5 µL, of the *P. falciparum* or *P. vivax* sample, using a thin blood smear to determine % parasitaemia and then converted to parasite concentration. The smear was fixed onto a glass slide using methanol and stained with Wright-Giemsa for 15 min. Parasites were visualized and counted using a 100 × oil immersion objective under white light. At least 500 RBC’s were counted to determine % parasitaemia. The Eq. (3) shows the calculation for estimating parasite concentration from the blood smear using an estimated average red cell count of 5,000,000 RBC’s per µL.

3 $$ Parasites/\mu L = \frac{{\# {\mkern 1mu} of{\mkern 1mu} parasites}}{{\# of{\mkern 1mu} RBC^{'}s}} \times 5{,}000{,}000 $$

Parasite densities for clinical samples were calculated based on the number of asexual parasites per µL of blood on a thick smear stained with 10% Giemsa. Parasites were counted until the field containing the 200th white blood cell (WBC) was reached. Then, density was calculated based on the study participant’s WBC count as described by Eq. (4) below:

4 $$ \frac{{\# \,{{of}}\,{{parasites}} \times {{WBC}}{\mkern 1mu} \,{{count}}}}{{\# {\mkern 1mu} \,{{of}}\,{\mkern 1mu} {{WBCs}}{\mkern 1mu} \,{{counted}}}} = \,\# \,o{{f}}\,{{parasites}}{\mkern 1mu} \,{{per}}\,{\mkern 1mu} \mu {{l}}\,{\mkern 1mu} {{of}}\,{\mkern 1mu} {{blood}} $$

### Statistical analysis

Statistical tests were used for data analysis of all specificity and sensitivity measurements. The LOD was determined from PD data by using a one-way ANOVA post-hoc Dunnett’s compared to the negative controls (NTC) with a 95% confidence interval. Box-and-whisker plots were made for PD measurements at the tenfold dilutions where the minimum and maximum values were represented by the upper and lower whiskers. Quartiles 25% and 75% were represented by the upper and lower bounds respectively. All graphs and analysis were performed using Graph Pad Prism 7.

## Results

### PD-LAMP comparison in phone and microscope

**Fig. 2:**
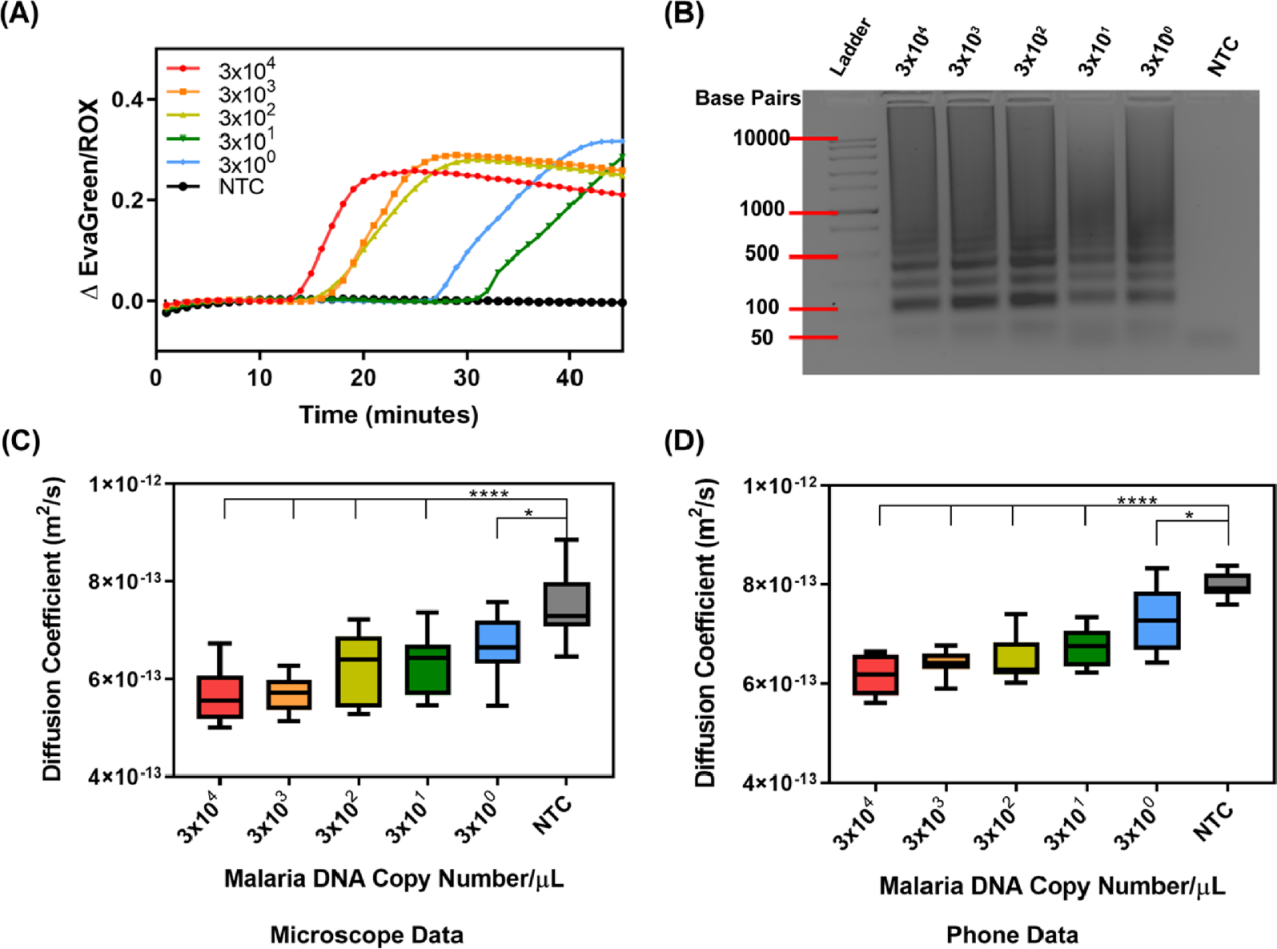
*Plasmodium falciparum* genomic DNA detection targeting 28 s rRNA. **A** Real-time fluorescence data during a 45-min LAMP reaction with concentrations ranging from 3 × 10^4^ to 3 × 10^0^ DNA copies/µL. **B** The representative LAMP DNA banding pattern for all positive samples are confirmed in 2% agarose gel electrophoresis (note the NTC shows no banding pattern). **C** PD analysis from the microscope images of the samples indicates the change in diffusion coefficient for LAMP samples with a statistically significant difference from the negative template control (NTC) for samples with 3 × 10^4^–3 × 10^1^ (****p < 0.0001) and 3 × 10^0^ (*p < 0.05) DNA copies/µL. **D** PD analysis on smartphone device indicates statistically significant differences between 3 × 10^4^ and 3 × 10^1^ (****p < 0.0001) and 3 × 10^0^ (*p < 0.05) DNA copies/µL and the NTC. NTC here represents water added in p lace of genomic DNA (N = 4)

LAMP reactions targeting the 28 s rRNA gene were performed across tenfold serial dilutions from 3 × 10^4^ to 3 × 10^0^ DNA copies/µL of *P. falciparum* DNA. All dilutions amplified in less than 45 min as visualized by the sigmoidal increase in real- time fluorescence measurements (Fig. 2A). Real-time fluorescence visualization shows that the highest initial concentrations of 28 s RNA amplified more rapidly. Negative template control (NTC) samples remained at baseline throughout the 45-min amplification for all instances in the qPCR graphs (N = 4) (Additional file 1: Figure S1). Amplification was confirmed with a 2% agarose gel showing banding only in positive samples (Fig. 2B).

Products of the 28 s rRNA LAMP assays were used to validate the PD measurements made on an inverted epifluorescent microscope against PD measurements on the smartphone device with an in-house MATLAB code. After performing a one-way ANOVA with Dunnett’s post-hoc against the NTC, we found that there were statistically significant differences between sample dilutions 3 × 10^4^ and 3 × 10^1^ (****p < 0.0001) and 3 × 10^0^ (*p < 0.05) DNA copies/µL relative to the NTC for both PD measurements on the microscope (Fig. 2C) and the smartphone device (Fig. 2D). PD yielded lower diffusion coefficients in positive samples as expected due to the inhibition of particle Brownian motion in the presence of malaria DNA amplicons. To ensure reproducibility each sample was measured in duplicate on each platform after four different amplification experiments (N = 4). There were no significant difference in the measurement efficacy between the microscope and smartphone platform (compare Fig. 2C and D).

### 28 s rRNA PD-LAMP specificity in blood

As *Plasmodium* parasites reside in red blood cells, we needed to assess the feasibility of PD-LAMP in whole blood samples. LAMP with *P. falciparum* genomic DNA was performed at a concentration of 10^4^ copies/µL in reactions containing several whole blood concentrations (v/v). The amplification time was extended from 45 to 60 min due to the inhibition caused by the addition of blood. Samples containing *P. falciparum* genomic DNA amplified when up to 10% of the reaction volume consisted of blood (Additional file 1: Figure S2). No amplification occurred in 15% blood or greater. Further, no non-specific amplification occurred with the NTC in the reactions consisting of 10% blood (Additional file 1: Figure S3). Therefore, 10% blood was the greatest concentration that could be used without inhibiting LAMP or causing non-specific amplification of control samples.

**Fig. 3:**
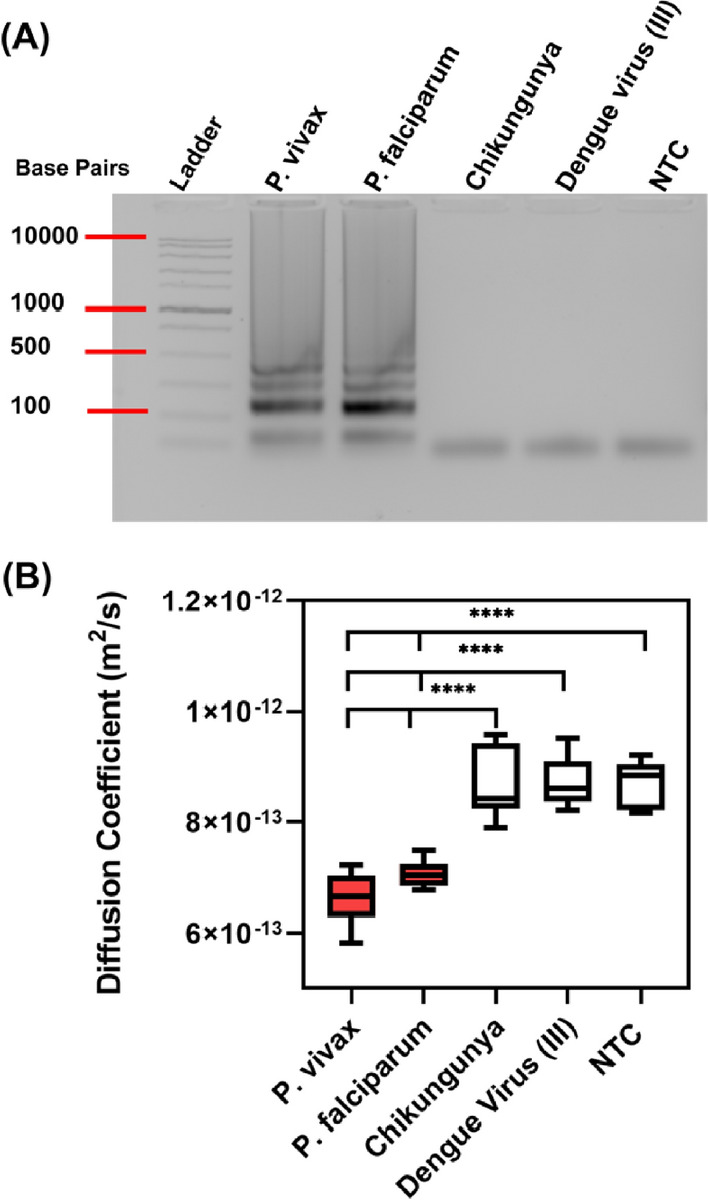
PD-LAMP specificity in 10% blood. **A** A 2% agarose gel from LAMP reactions in blood containing malarial strains *P. falciparum* and *P. vivax* alongside controls Chikungunya and dengue virus (III) at 6 × 10^4^ parasites/µL blood after a 60-min LAMP reaction. Only malaria samples amplified, demonstrated by the DNA banding pattern in the gel. **B** Diffusion coefficients from smartphone PD analysis, where malaria samples showed a significant difference from Chikungunya virus, dengue virus (III) and NTC (One-way ANOVA Dunnett’s post-hoc test). NTC represents blood without spiked pathogens (N = 3)

To ensure assay selectivity for malaria, LAMP targeting 28 s rRNA was performed with *P. falciparum* and *P. vivax* blood samples (BEI), alongside dengue virus (III) and Chikungunya virus RNA, which are also mosquito-borne pathogens, but do not contain the 28 s rRNA gene. A 60-min LAMP assays were performed for each sample in a reaction containing 10% blood. Specific amplification occurred for the *P. falciparum* and *P. vivax* DNA samples, while dengue virus (III) and Chikungunya virus did not amplify, as indicated on an agarose gel (Fig. 3A). Following amplification, we performed PD on the LAMP samples. Dunnett’s post-hoc test was used against each individual sample. There was no significant difference in the PD signal relative to the NTC (p > 0.5) in the presence of dengue virus (III) or Chikungunya virus RNA (N = 4) (Fig. 3B). Alternatively, both malaria positive samples (*P. falciparum* and *P. vivax*) were found to be significantly different from dengue and Chikungunya virus samples and the NTC (uninfected blood) (****p < 0.0001), but not from each other.

### PD-LAMP sensitivity in 10% blood (v/v) with infected RBCs

**Fig. 4:**
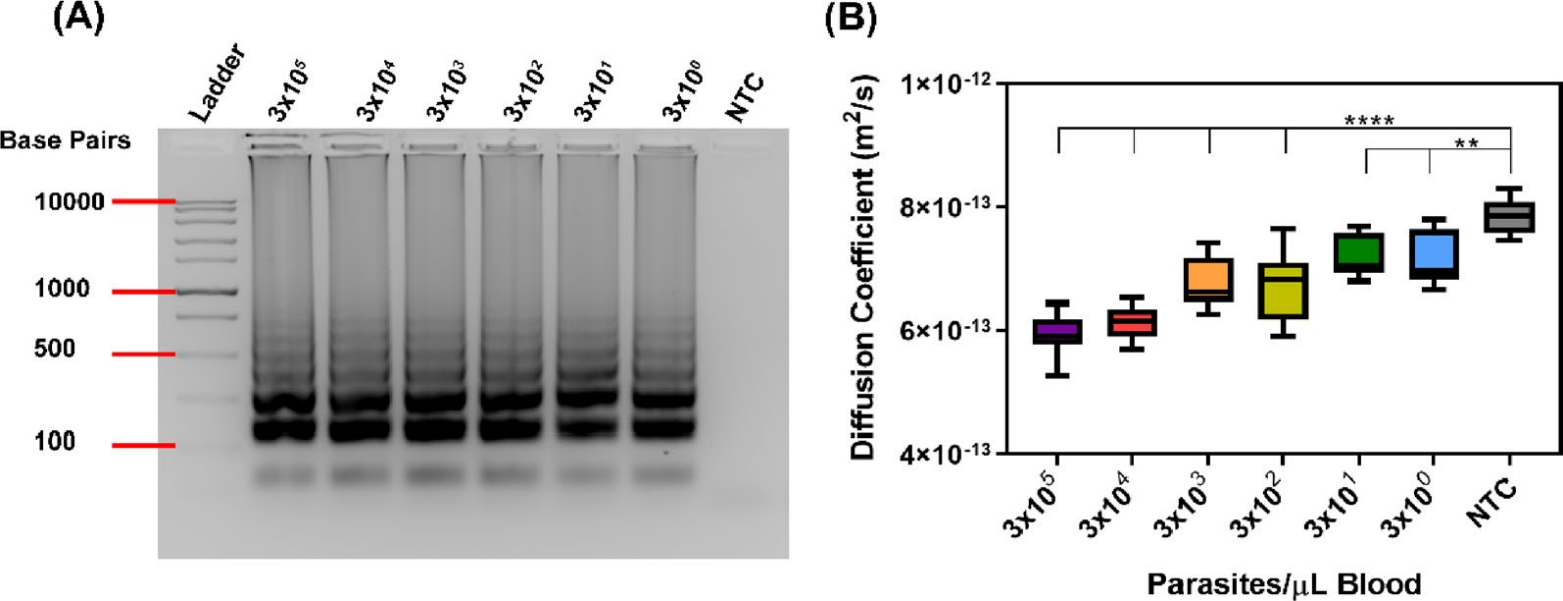
Sensitivity of malaria PD-LAMP with 28 s primers using 10% blood. **A** 2% agarose gel confirming amplification in positive samples. **B** Diffusion coefficients measured using smartphone device for dilutions of 3 × 10^5^–3 × 10^0^ of *P. falciparum* infected blood after a 75-min reaction. PD analysis shows statistical difference from controls for 3 × 10^5^–3 × 10^2^ parasites/µL blood (****p < 0.0001) and 3 × 10^1^–3 × 10^0^ parasites/µL blood (**p < 0.001) (N = 4)

The LOD of PD-LAMP in 10% blood was determined using commercially available malaria infected blood samples. The stock infected blood was diluted with uninfected blood to obtain tenfold dilutions between 3 × 10^5^ and 3 × 10^0^ parasites/µL of blood for a 75-min LAMP reaction using 28 s rRNA primers. The LOD of the LAMP assay was 3 parasites/µL. Amplification was confirmed with a 2% agarose gel (Fig. 4A). Further, when measured by PD-LAMP, amplification from as few as 3 parasites/µL blood resulted in a significantly reduced diffusion coefficient compared to NTC in blood (N = 4) (Fig. 4B). Dunnett’s post-hoc test for all samples confirmed significance from NTC with ****p < 0.0001 for 3 × 10^5^ to 3 × 10^2^ and **p < 0.001 for 3 × 10^1^ to 3 × 10^0^.

### Blinded study of PD-LAMP in infected blood samples with PCR confirmation

A blinded study using patient blood samples was used to validate the robustness of the smartphone device. Six previously collected and de-identified malaria samples (labelled patient ID 1–6) with parasite densities ranging from 4 to 265,782 parasites/µL as quantified by qPCR were used for this study [44]. Each sample was analysed on the smartphone via PD-LAMP in a blinded study where the user of the device did not know the nature of the samples being analysed. The samples were amplified using two different primer sets, 28 s rRNA and 18 s rRNA. The 18 s rRNA primer set was introduced to this work to reduce the amplification time of the patient samples because there are more copies of the 18 s rRNA gene than 28 s rRNA in the *P. falciparum* genome [48]. Table 1 shows qPCR values, microscopy, and diffusion coefficients (PD-LAMP) for each sample using the 18 s and 28 s rRNA primers. The 18 s rRNA PD-LAMP resulted in diffusion coefficients of less than 7.2e^−13^ m^2^/s for all patients with parasitaemia while the NTC resulted in a diffusion coefficient of 9.0e^−13^ m^2^/s, higher than the positive samples.

**Table 1:** Parasite densities and average diffusion coefficients from 18 and 28 s rRNA primers of patient samples. Quantitative PCR and PD-LAMP was performed on six infected blood samples and a negative control (uninfected blood). A significant difference (p < 0.05) was shown in the

Patient ID	Group	Parasite density by microscopy (parasites/µL)	Parasite density by qPCR (parasites/µL)	28 s diffusion coeff. (± standard dev.) (m^2^/s)	18 s diffusion coeff. (± standard dev.) (m^2^/s)
1	UM^a^	N.D.^e^	265,782	6.4(± 0.51)e^−13^	6.1(± 0.61)e^−13^
2	CM^b^	64	126	6.7(± 1.31)e^−13^	7.0(± 0.58)e^−13^
3	SMA^c^	524^d^	4	7.9(± 0.91)e^−13^	6.9(± 0.78)e^−13^
4	CM	545	2552	7.7(± 0.67)e^−13^	6.9(± 0.28)e^−13^
5	CM	26,643	1466	6.5(± 0.51)e^−13^	7.1(± 0.45)e^−13^
6	CM	511,972	N.D.	7.05(± 0.25)e^−13^	6.9(± 0.35)e^−13^
NTC	–	0	–	8.8(± 0.73)e^−13^	9.0(± 1.01)e^−13^

^a^Uncomplicated malaria

^b^Cerebral malaria

^c^Severe malaria anaemia

^d^Parasite density not corrected for white blood cell count

^e^Not determined

From the LAMP assay, all six patient samples that incorporated primers targeting the 18 s rRNA gene amplified within 45 min. Samples with the 28 s rRNA primer set underwent a 90-min reaction and two of the patient samples did not amplify. Agarose gel electrophoresis confirmed LAMP amplification of all positive samples at the end of the blinded study using the 18 s primer set (N = 3) (Additional file 1: Figure S5A). Diffusion coefficient values, measured with PD, for the patient samples were significantly different from NTC for 18 s rRNA gene target (****p < 0.0001) (Additional file 1: Figure S5B). It was observed that samples from Patient ID 3 and 4 both showed inconsistent amplification in the agarose gels while all other patient samples showed consistent amplification between repeats (N = 3) (Additional file 1: Figure S5C). Targeting the 28 s rRNA gene, patient ID 3 and 4 were not found to be significantly different from NTC after a 90-min amplification. All other patient samples with LAMP targeting the 28 s rRNA gene were found to be significant from PD measurements (****p < 0.0001 for 1 and 5, ***p < 0.001 for 2, **p < 0.01 for 6) (Additional file 1: Figure S5D). The nature of this blinded study showed reproducibility in using PD for the detection of LAMP amplicons in the smartphone device as well as the importance of choosing a robust LAMP assay target.

### LOD from diluted patient samples

**Fig. 5:**
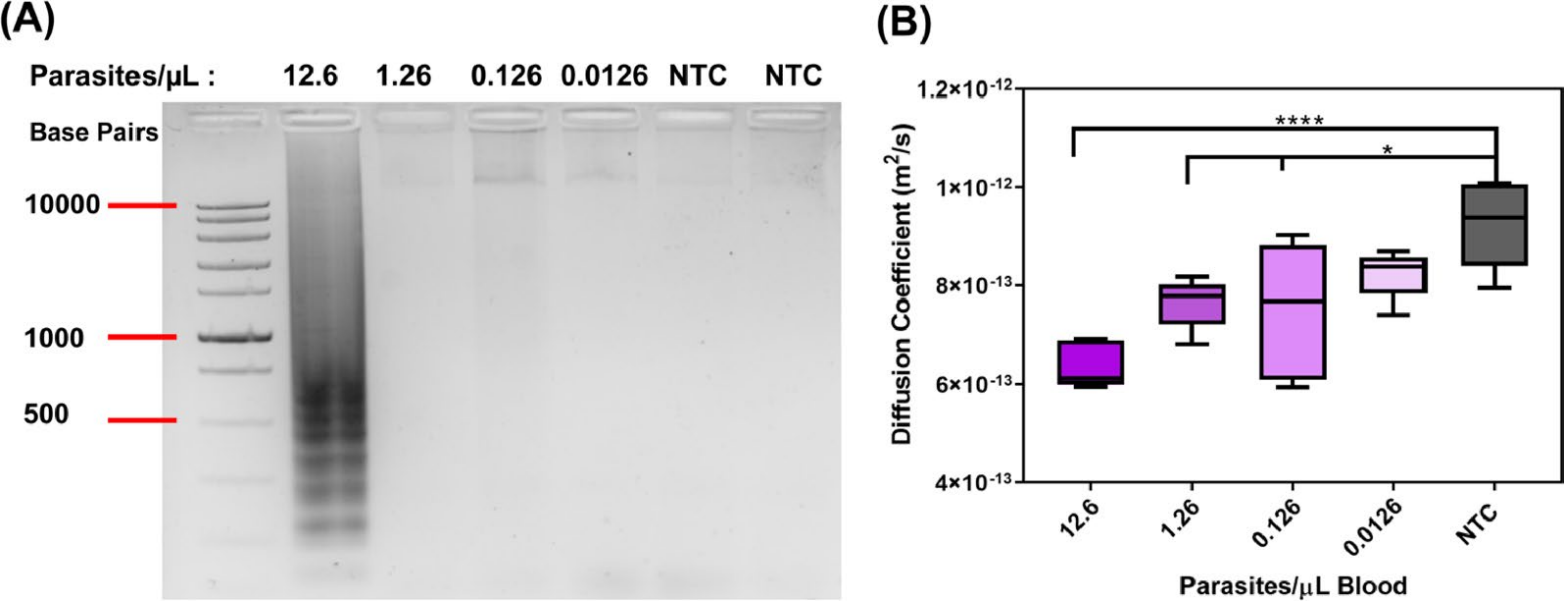
Serial dilutions of patient ID 2 for the determination of the LOD in patient samples using 18 s primers. **A** A representative gel from dilutions of ID 2 with concentrations ranging from 12.6 to 0.0126 parasites/μL. Only the 12.6 parasites/μL sample showed consistent amplification in gel represented by the dark smeared banding. **B** Diffusion coefficients from smartphone PD analysis of diluted samples yielded a LOD of 0.126 parasites/µL blood. All dilutions, 12.6 parasites/µL blood (****p < 0.0001), 1.26 and 0.126 parasites/µL blood (*p < 0.05), except 0.0126 parasites/µL blood were found to be statistically different from NTC. (N = 3)

Patient samples were used to determine the LOD of PD-LAMP on the smartphone device. Dilutions from sample ID 2, the 2nd lowest concentration by qPCR, were performed in purchased blood starting with a dilution from stock of 12.6 to 0.0126 parasites/µL and performed PD after a 45-min LAMP reaction using the 18 s rRNA primer set. As shown in Additional file 1: Figure S5, the 18 s rRNA primer set had greater reproducibility and amplified in less time than the 28 s rRNA primer set. Therefore, 18 s rRNA primer sets were chosen for this sensitivity study. The 2% agarose gel indicated inconsistent amplification between repeats below 12.6 parasites/µL (Fig. 5A). However, the LOD of PD-LAMP on the smartphone device was found to be 0.126 parasites/µL (Fig. 5B). The diffusion coefficient values were significantly different from NTC for 12.6 (****p < 0.0001), 1.26 and 0.126 (*p < 0.05) parasites/µL blood using one-way ANOVA post-hoc Dunnett’s test. The lowest concentration, 0.0126 parasites/µL blood, was not significantly different from NTC (uninfected blood) but followed the same trend of having lower diffusivity than the NTC. Thus, PD-LAMP is sensitive enough to detect amplicons even when they cannot be ascertained via agarose gel electrophoresis and a LOD of 0.126 parasites/µL was obtained with the smartphone device from patient samples.

### PD-LAMP from asymptomatic samples

To determine if PD-LAMP could accurately detect *P. falciparum* in asymptomatic study participants, whole blood samples were collected from 11 healthy asymptomatic individuals (community controls, CC) and two symptomatic individuals (Cerebral Malaria, CM and Severe Malaria Anaemia, SMA) from Uganda and analysed via microscopy on site and were stored for nPCR, qPCR, and PD-LAMP testing. Samples were tested in duplicate for qPCR assays targeting *var*ATS gene, and for PD-LAMP assays using the 18 s rRNA gene (N = 4) (Table 2). For qPCR, the sample was deemed negative if all replicates were negative. Each sample was also run on an agarose gel after a 45-min LAMP reaction. Six of the 11 asymptomatic samples to be positive for *P. falciparum* via PD-LAMP using Dunnett’s multiple comparison test to healthy parasite-free controls, the same was true for the two out of two symptomatic individuals*.* PD-LAMP positive results correlated with 8/9 of the positive results by qPCR. Therefore, PD-LAMP was 89% sensitive and 100% specific when compared to qPCR (Table 2). The nPCR, targeting 18 s rRNA gene, detected two more positive cases than PD-LAMP or qPCR (Table 2). This discordance among molecular methods has been documented in samples with very low levels of parasitaemia [49–51].

**Table 2:** Presence of *P. falciparum* parasitaemia as assessed by nPCR, qPCR and PD-LAMP in community children with asymptomatic parasitaemia (CC), and positive control children with cerebral malaria (CM) or severe malarial anaemia (SMA)

Study ID	Group	Parasite presence (nPCR)	Parasite density by qPCR (parasites/µL)	PD-LAMP
7	CC^a^	−	0	−
8	CC	−	1	+
9	CC	−	0	−
10	CC	+	0	−
11	CC	+	0	−
12	CC	+	1	−
13	CC	+	56	+
14	CC	+	35	+
15	CC	+	12,749	+
16	CC	+	8.741	+
17	CC	+	100,669	+
18	CM^b^	N.D.^d^	377,406	+
19	SMA^c^	N.D.^d^	136,926	+

− = negative for *P. falciparum* DNA, + = positive for *P. falciparum* DNA

^a^Community controls

^b^Cerebral malaria

^c^Severe malaria anaemia

^d^Not determined

## Discussion

Many low and middle income countries (LMICs) struggle to maintain laboratory-intensive testing programs that are necessary for accurate malaria diagnoses [4]. However, widescale testing for asymptomatic malaria infections will be a necessary component of WHO malaria reduction and elimination efforts [52]. In this work, PD-LAMP measurements were performed on a portable smartphone-enabled platform for the sensitive, rapid, and robust detection of malaria parasites from unfiltered blood. Genomic *P. falciparum* DNA was used to validate malaria detection on the smartphone first by comparing its results to PD measurements from a fluorescent microscope. The PD-LAMP smartphone platform has a LOD of 3 parasites/µL (Fig. 2), which is comparable to qPCR and Loopamp™ detection limits [23, 53]. Detection at this low concentration is promising for identifying asymptomatic cases that cannot be identified via microscopy. With this smartphone platform, PD-LAMP detects as few as 3 parasites/μL in 10% whole blood and is 66-fold more sensitive than currently used RDTs and comparable to the LAMP kit without sample purification [23].

Further, the PD-LAMP smartphone detection technique is sixfold more sensitive than even emerging ultrasensitive RDTs, which detect down to 20 parasites/μL [21, 54]. By directly adding blood to the LAMP reaction, the need for DNA extraction and pre-processing steps are eliminated (Fig. 4). These results demonstrate that the PD-LAMP smartphone device could be used for point-of-care malaria testing.

The selectivity of the pan*-Plasmodium* 28 s rRNA LAMP reaction to malaria was confirmed by testing against Chikungunya virus and dengue virus (III) RNA, spiked into blood. These viruses are also mosquito-borne and may have similar symptoms as malaria [55]. There are also some regions where there are coinfections of dengue or or Chikungunya with malaria [55]. Specifically identifying the *Plasmodium* parasite can aid in proper treatment amidst confounding symptoms. LAMP-mediated amplification and a resulting change in diffusivity only occurred in the positive malaria samples (Fig. 3). Additionally, the specificity of the LAMP assay was confirmed at various concentrations of blood, discovering that none of the negative controls amplified (Additional file 1: Figure S3). This proves that this malaria LAMP assay is specific to the *Plasmodium* genus.

PD-LAMP can also detect malaria at low concentrations from patient samples in 45 min (Table 1 and Table 2). Previous work has determined cut-off diffusion coefficients where below 7.3 × 10^–13^ are deemed positive and above 7.6 × 10^–13^ are negative [36]. Those that land in the middle would be deemed inconclusive and need retesting. It is expected the samples with 10% blood to follow this criteria (Table 1), but at least 100 samples are needed for confirmation. Of the first six patient samples tested, the 28 s rRNA primer set failed to amplify patient ID 3 and ID 4 consistently. The amplification inconsistencies with ID 3 and ID 4 may be due to the low copy number or low copies of the 28 s target gene. Conversely, the primer set targeting 18 s rRNA successfully amplified all six of the same patient samples within 45 min. Therefore, it was identified that the samples were likely not degraded over time, but rather the difference in gene target copies had a greater effect in ID 3 and ID 4. The use of multiple primer sets, such as 28 s and 18 s rRNA targeted primers, could prove useful for multiplexing and targeting multiple regions to combat emerging mutations in malaria DNA [56]. Although the sensitivity of the PD-LAMP device is not superior to the commercial Eiken kit, the methods developed in this work do not require pre-processing steps of the blood sample and works in complex sample matrices.

The PD-LAMP method was compared to a variety of well-established malaria detection methods. For example, detection of *P. falciparum* parasites from asymptomatic participants occurred with 89% sensitivity and 100% specificity when compared to qPCR performed on DNA from the same whole blood samples (Table 2). qPCR using DNA extracted from whole blood is comparable to the PD-LAMP method. However, nPCR detected two more positive cases than PD-LAMP or qPCR, which could also be a result of PCR irreproducibility at ultra-low parasite densities [49]. qPCR sensitivity is positively correlated with template copy number [51]. Low template numbers are subject to the “Monte Carlo” effect, where the success of a primer annealing and replicating during PCR is random [57]. In samples with high parasite densities, this effect is minimal since the template number is high, so the probability of primer binding, and ultimately replicating, is very high. But, in samples with low parasite densities, template copy number is lower, and the probability of binding and replication is much lower, resulting in reduced PCR yield and irreproducible results. The differences seen between nPCR, PD-LAMP and qPCR can be attributed to the difference in the targeted regions for amplification. There were variances in repeats for qPCR and PD-LAMP due to the low concentration of DNA, however PD-LAMP detected a positive sample at a concentration of 1 parasite/µL that was undetectable by microscopy.

The use of PD-LAMP on a smartphone is a promising technique for rapid detection of malaria at the point-of-care, because PD-LAMP eliminates the need for DNA extraction steps or the need to rely on antibody-antigen measurements. Ultimately, a smartphone-enabled hardware device could integrate a portable heating element for a standalone, sample-to-answer, portable diagnostic [26]. Total reaction volumes can also be increased to allow for higher input of blood sample while keeping the overall concentration of blood at 10%. Additionally, malaria PD-LAMP applications could also be extended for use with alternative sample matrices, such as dried blood spots and urine, which would provide users to have alternative sample storage and/or perform non-invasive screening. Future development of malaria PD-LAMP will involve field-testing in low-resource areas and multiplexing for detection of coinfections.

## Conclusion

In this work, detection of low concentrations of malaria DNA from unprocessed blood samples is enabled through the use of a smartphone-enabled device that is robust, portable, and has potential to be used in low-resource settings. Concentrations of 3 copies/µL from *P. falciparum* DNA were detected. However, larger studies will be required to determine the full sensitivity and specificity of these techniques. Of the asymptomatic samples tested (Table 2) all but one that was detected by qPCR was detected by PD-LAMP yielding 89% sensitivity and 100% specificity. From the 13 samples tested by qPCR (Table 2) and PD-LAMP, the concordance was 92%. One sample that was positive for both nPCR and qPCR was negative for PD-LAMP. This observation was not entirely surprising since reproducibility of DNA amplification (regardless of DNA amplification method) in samples with very low parasitaemia may present alternating positive and negative results in 38% of the testing [49]. It is also of note that PD-LAMP was able to detect two more samples that were undetectable by gold standard microscopy (Additional file 1: Table S5).

This work demonstrates that *Plasmodium* parasites can be detected from whole blood specifically and robustly with PD-LAMP at concentrations down to 1 parasite/µL with no need for DNA extraction or pre-processing. Further, *P. falciparum* parasites from asymptomatic participants were detected with 89% sensitivity and 100% specificity when compared to qPCR measurements from the same samples. This sets the stage for obtaining a larger amount of clinical samples in future studies.

Current diagnostics are unable to rapidly and accurately detect parasitaemia below 100 parasites/µL, which is one reason why there has been poor progress toward the reduction of malaria transmission [58]. The sensitivity of the PD-LAMP device is competitive against field-based testing techniques such as RDTs, Loopamp™ malaria kit, and white light microscopy. Future work includes incorporating dried reagents on-chip for long term storage and integrating heating into the device to perform the assays all on one handheld platform. Ultimately, a fully integrated PD-LAMP smartphone device could improve public health in malaria endemic areas through rapid low parasitemia detection and aid in process towards eradication of the infectious disease.

## Supplementary Information

**Additional file 1: Figure S1.** Real-time fluorescence and PD-LAMP measurements for each individual repeat. **Figure S2.** Positive ( +) (3 × 10^4^) and negative (-) samples (molecular biology water) at blood concentrations of 0–10% (v/v). **Figure S3.** Specificity of malaria PD-LAMP in 0, 5 and 10% blood (v/v). **Figure S4.** Specificity of malaria PD-LAMP for 18 s rRNA in 10% blood. **Figure S5.** PD-LAMP performed on patient malaria samples. **Figure S6.** Dot Plot of patient samples from blinded study. Diffusion coefficients of patient samples with varying parasite densities using the 28 s primer set. **Table S1**. Nucleotide Sequences for LAMP Primers Targeting 28srRNA (Bio = biotin). **Table S2.** Nucleotide Sequences for LAMP Primers Targeting 18srRNA (Bio = biotin). **Table S3.** LAMP Master Mix Used for Amplification of Malaria DNA. **Table S4.** Oligonucleotide Sequences and qPCR Conditions for *var*ATS Assays. **Table S5.** Asymptomatic Individual Sample Groups and Amplification Results. **Figure S7**. Representative Images of 400 nm streptavidin-coated particles in a LAMP sample. **Figure S8.** Chip Schematic. Layers of 188 µm COP are heat pressed together and hole punched. Double sided PSA is used to form a well for the sample. **Figure S9.** Image of Smartphone device and chip.

**Additional file 2: Video S1.** Representative clip from a positive PD-LAMP sample taken on the smartphone device.

**Additional file 3: Video S2**. Representative clip from a negative PD-LAMP sample taken on the smartphone device.

## Data Availability

The datasets used and/or analysed during the current study are available from the corresponding author on reasonable request.
