# Supplementary material for: Towards the use of a smartphone imaging-based tool for point-of-care detection of asymptomatic low-density malaria parasitaemia
Source: Malar J. 2021 Sep 25;20:380. doi: 10.1186/s12936-021-03894-w (PMC8466697; doi:10.1186/s12936-021-03894-w)
Supplement: Supplementary file 1 — Additional file 1: Figure S1. Real-time fluorescence and PD-LAMP measurements for each individual repeat. Figure S2. Positive ( +) (3 × 10^4^) and negative (-) samples (molecular biology water) at blood concentrations of 0–10% (v/v). Figure S3. Specificity of malaria PD-LAMP in 0, 5 and 10% blood (v/v). Figure S4. Specificity of malaria PD-LAMP for 18 s rRNA in 10% blood. Figure S5. PD-LAMP performed on patient malaria samples. Figure S6. Dot Plot of patient samples from blinded study. Diffusion coefficients of patient samples with varying parasite densities using the 28 s primer set. Table S1. Nucleotide Sequences for LAMP Primers Targeting 28srRNA (Bio = biotin). Table S2. Nucleotide Sequences for LAMP Primers Targeting 18srRNA (Bio = biotin). Table S3. LAMP Master Mix Used for Amplification of Malaria DNA. Table S4. Oligonucleotide Sequences and qPCR Conditions for varATS Assays. Table S5. Asymptomatic Individual Sample Groups and Amplification Results. Figure S7. Representative Images of 400 nm streptavidin-coated particles in a LAMP sample. Figure S8. Chip Schematic. Layers of 188 µm COP are heat pressed together and hole punched. Double sided PSA is used to form a well for the sample. Figure S9. Image of Smartphone device and chip. [file 12936_2021_3894_MOESM1_ESM.docx]

# **Towards the use of a smartphone imaging-based tool for point-of-care detection of asymptomatic low-density malaria parasitaemia**

Ashlee J. Colbert^1^, Katrina Co^2^, Giselle Lima-Cooper^2^, Dong Hoon Lee^3^, Katherine N. Clayton^4^, Steven T. Wereley^3^, Chandy C. John^2^, Jacqueline C. Linnes^1*^, Tamara L. Kinzer-Ursem^1*^

^1^Weldon School of Biomedical Engineering, Purdue University, West Lafayette, IN, 47907, USA

^2^Indiana University School of Medicine, Indiana University, Indianapolis, IN, 46202, USA

^3^School of Mechanical Engineering, Purdue University, West Lafayette, IN, 47907, USA

^4^OmniVis Inc., Indianapolis, IN, 46201, USA

*Corresponding authors

Email: jlinnes@purdue.edu; tursem@purdue.edu

**Keywords** Malaria, particle-diffusometry, nucleic-acid based tests, smartphone-detection, LAMP-assay

**Additional file 1:**


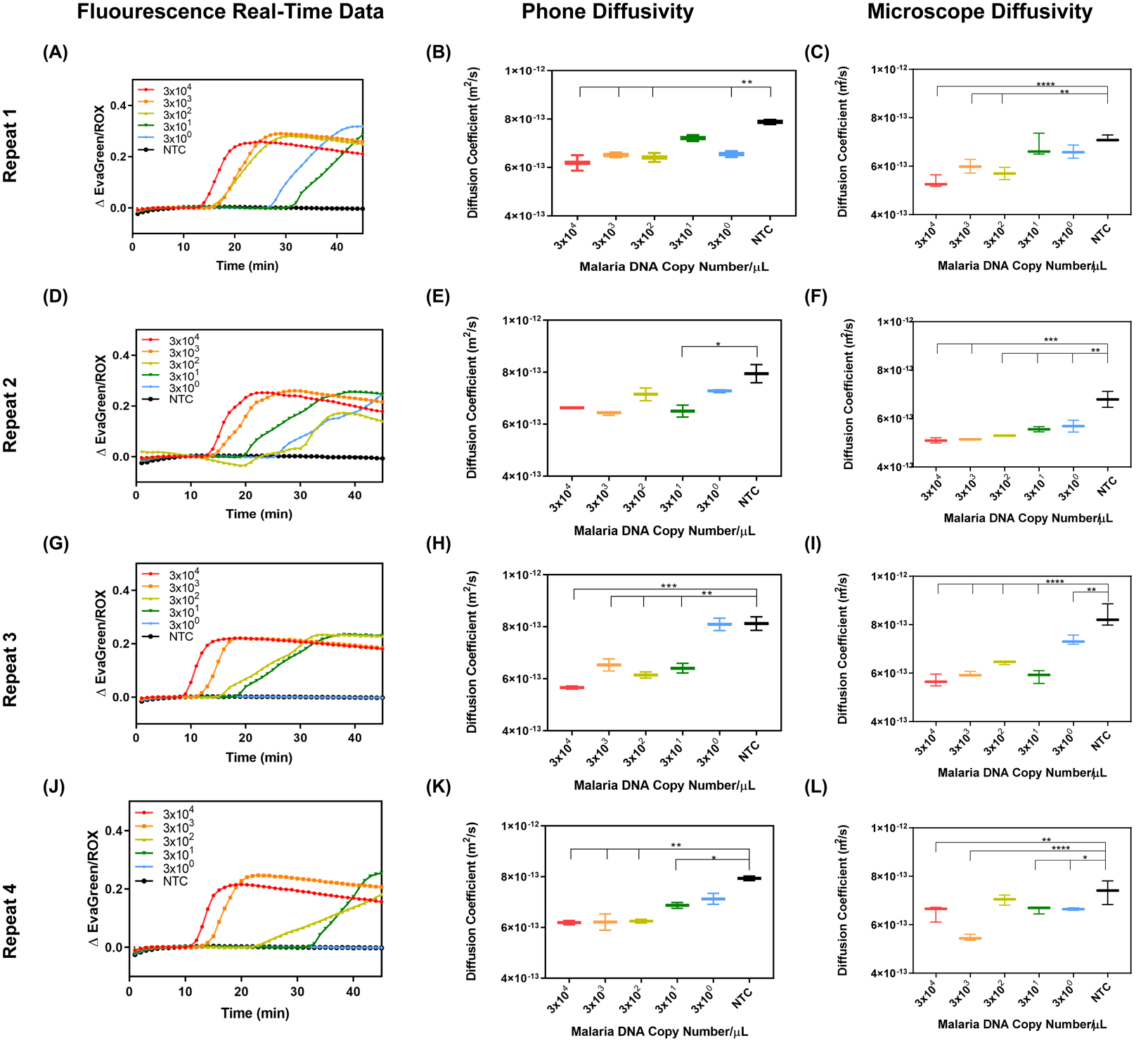
**Figure S1.** Real-time fluorescence and PD-LAMP measurements for each individual repeat. (A) Real-time fluorescence curves for DNA amplification of varying concentrations for repeat 1. (B) PD measurements performed on smartphone for same samples in repeat 1 with significant difference from control for 3x10^4^, 3x10^3^, 3x10^2^, and 3x10^0^ (**p<0.01). (C) PD measurements performed on microscope for all samples in repeat 2 with significant differences from control for 3x10^4(^****p<0.0001), 3x10^3^ and 3x10^2^ (***p<0.001). (D) Real-time fluorescence curves for DNA amplification of varying concentrations for repeat 2. (E) PD measurements performed on smartphone for same samples in repeat 2 with significant difference from control for 3x10^1^(*p<0.05). (F) PD measurements performed on microscope for all samples in repeat 2 with significant differences from control for 3x10^4^, 3x10^3^ (***p<0.001), 3x10^2^, 3x10^1^, and 3x10^0 (^**p<0.01). (G) Real-time fluorescence curves for DNA amplification of varying concentrations for repeat 3. (H) PD measurements performed on smartphone for same samples in repeat 3 with significant difference from control for 3x10^4^ (***p<0.001), 3x10^3^, 3x10^2^, and 3x10^1^ **(p<0.01). (I) PD measurements performed on microscope for all samples in repeat 3 with significant differences from control for 3x10^4^, 3x10^3^, 3x10^2^, 3x10^1^ (****p<0.0001), and 3x10^0 (^**p<0.01). (J) Real-time fluorescence curves for DNA amplification of varying concentrations for repeat 4. (K) PD measurements performed on smartphone for same samples in repeat 4 with significant difference from control for 3x10^4^, 3x10^3^, 3x10^2^ (***p<0.001), and 3x10^1^ *(p<0.05). (L) PD measurements performed on microscope for all samples in repeat 4 with significant differences from control for 3x10^4^(**p<0.01), 3x10^3^(****p<0.0001), 3x10^1^ and 3x10^0 (^*p<0.05).


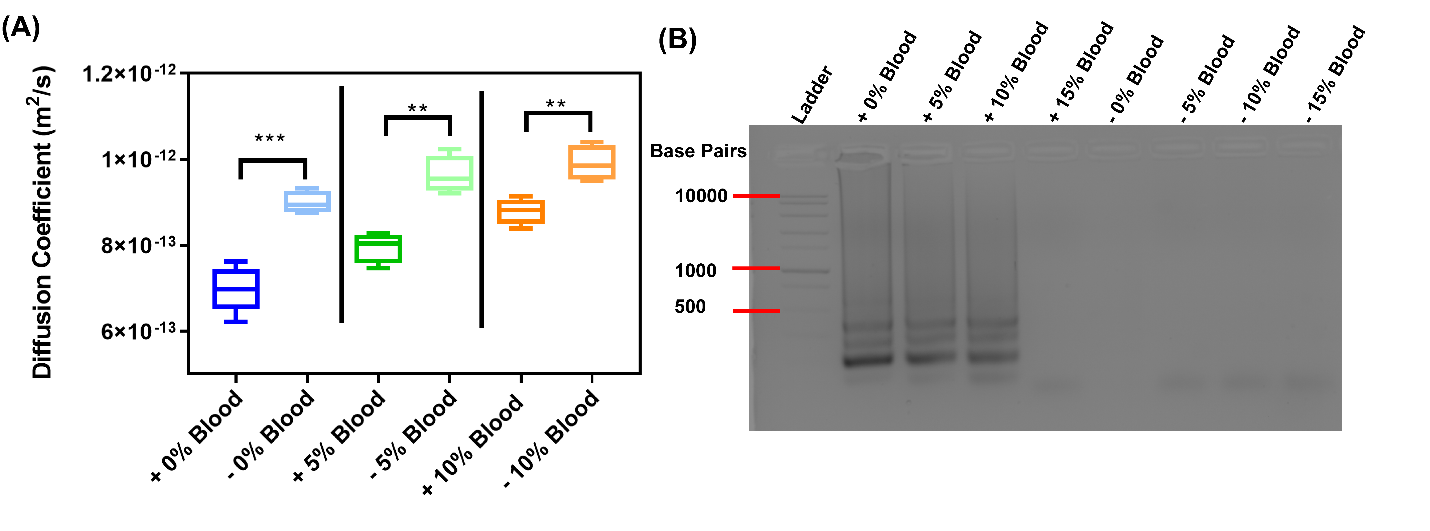
**Figure S2.** Positive (+) (3x104) and negative (-) samples (molecular biology water) at blood concentrations of 0-10% (v/v) show a significant difference from controls at each concentration (**p<0.01, ****p<0.0001) using a t-test (A). The 2% agarose gel confirms that there was no amplification in 15% blood (no banding pattern) and no negatives amplified (N=3) (B).


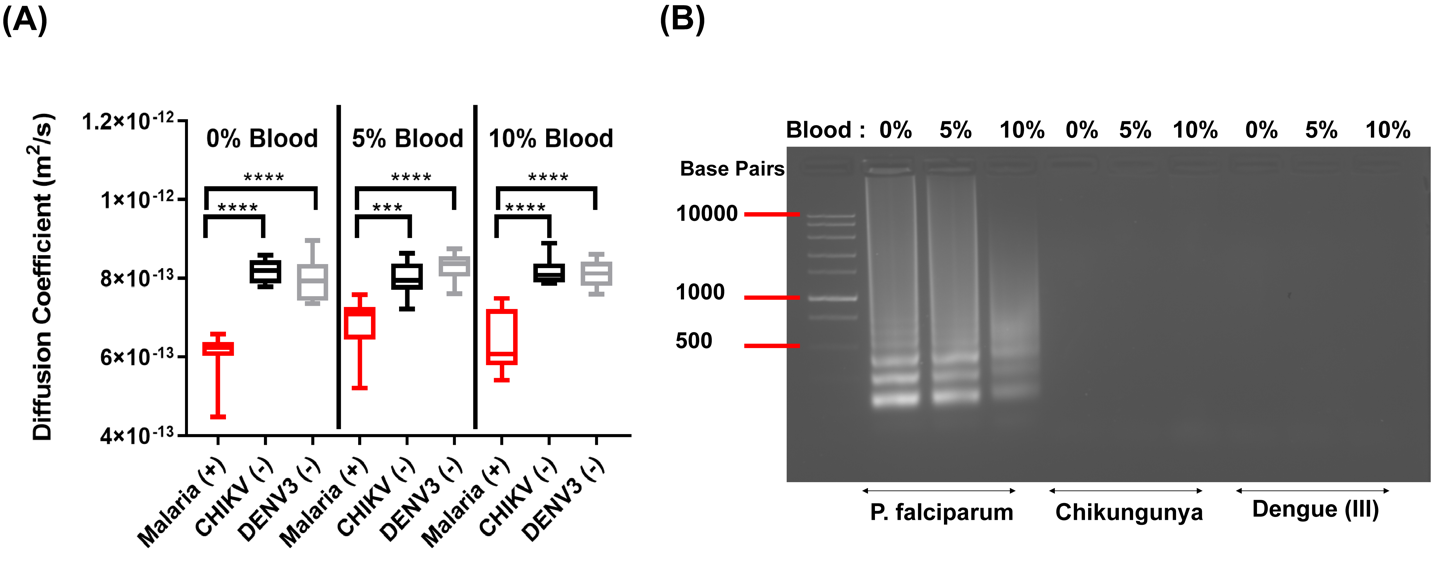


**Figure S3.** Specificity of malaria PD-LAMP in 0, 5 and 10% blood (v/v). (A) PD diffusion coefficients at each concentration were significantly different from chikungunya (CHIKV) and dengue virus Type 3 (DENV3) controls (***p<0.0001). (B) 2% agarose gel confirmation of amplification for only positive reactions identified by characteristic LAMP DNA banding. All DNA concentrations used were 3x104 DNA copies/µL. Malaria DNA used here was P. falciparum.


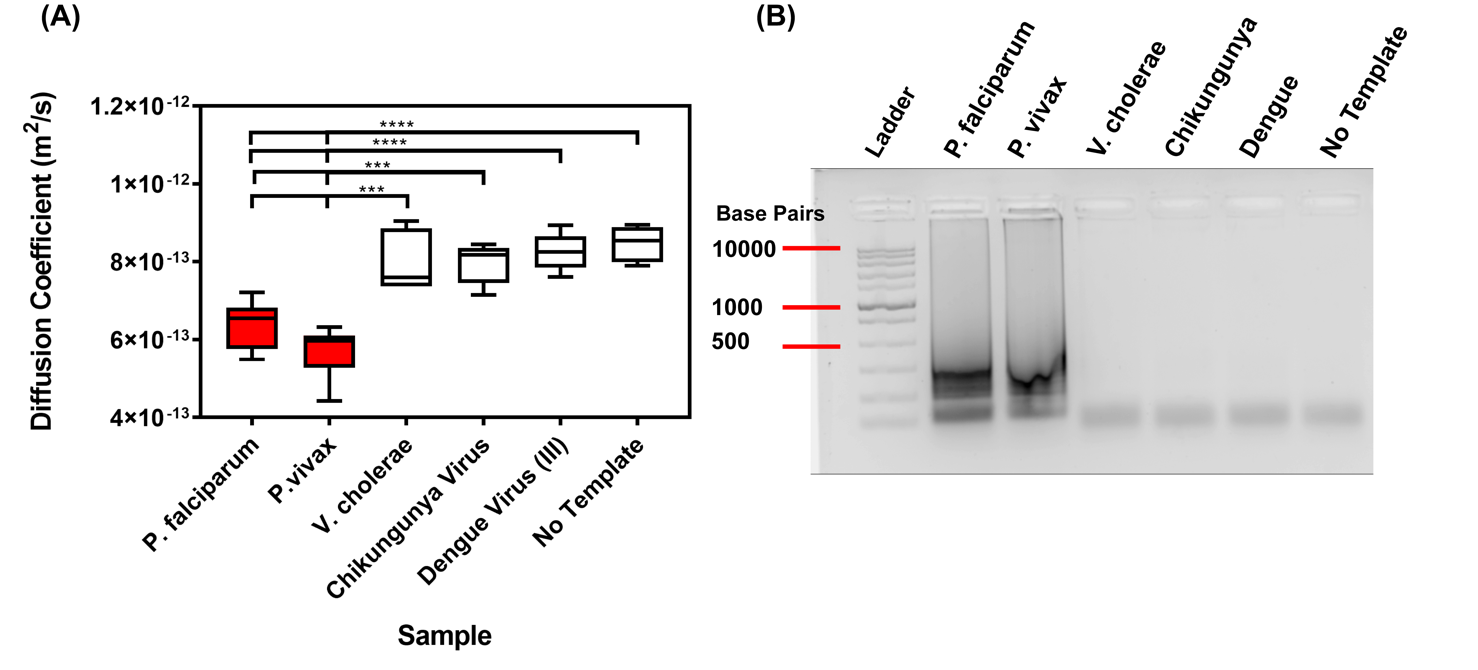


**Figure S4.** Specificity of malaria PD-LAMP for 18s rRNA in 10% blood. (A) PD Diffusivity coefficients at each concentration significantly different from each control (***p<0.0001). (B) Representative agarose gel to confirm presence of banding only in malaria samples. (N=4)

**
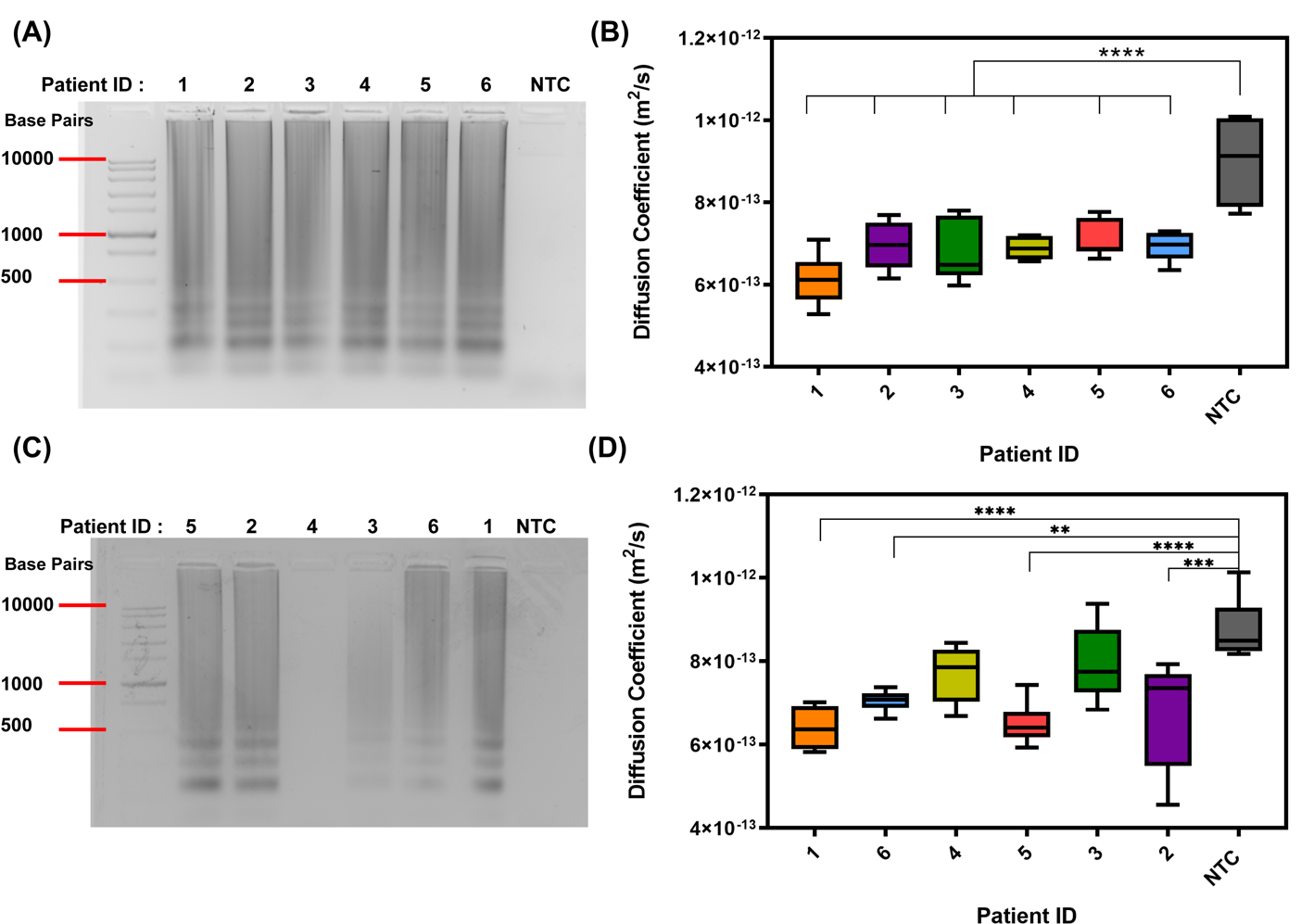
**

28s rRNA

18s rRNA

**Figure S5.** PD-LAMP performed on patient malaria samples. (A) 2% agarose gel confirmation of amplification in all patient samples using 18s rRNA primers. (B) Diffusion coefficients of patient samples with varying parasite densities after a 45-minute reaction (18s rRNA). PD analysis shows all patient samples were significantly different from the NTC (****p<0.0001). (C) A representative 2% agarose gel after a 90-minute LAMP reaction (28s rRNA) indicated amplification in all samples except patient ID 4 and faint amplification in patient ID 3. (D) Diffusion coefficients of patient samples with varying parasite densities after a 90-minute reaction (28s rRNA). PD analysis showed a statistical significance from NTC for patient IDs 1, 5 (****p<0.0001), 2 (***p<0.001), and 6 (**p<0.01). N=3.


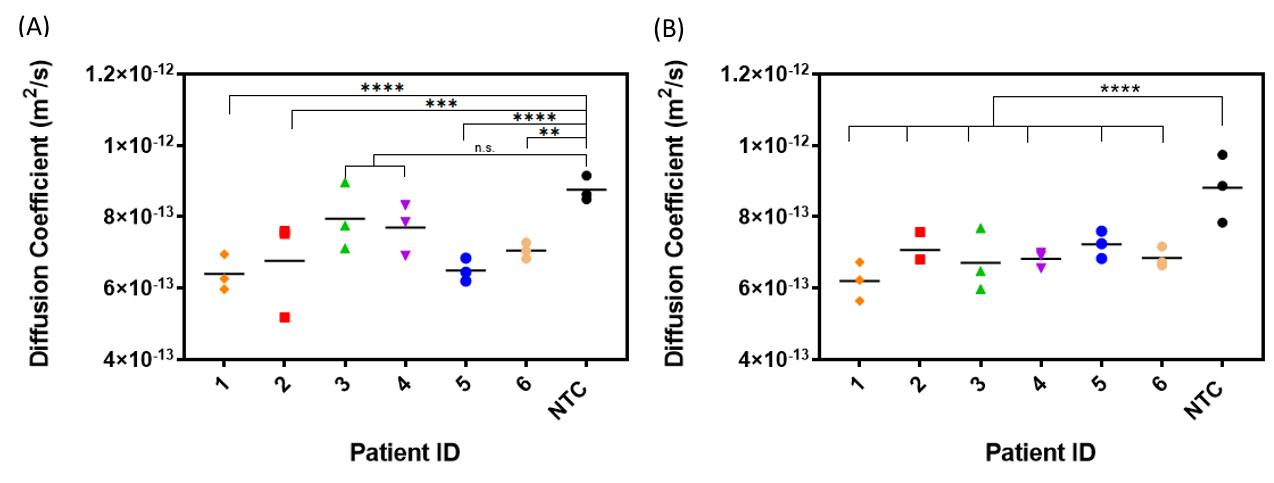


**Figure S6.** Dot Plot of patient samples from blinded study. Diffusion coefficients of patient samples with varying parasite densities using the 28s primer set. PD analysis showed a statistical significance from NTC for patient IDs 1, 5 (****p<0.0001), 2 (***p<0.001), and 6 (**p<0.01). N=3.(B) Diffusion coefficients averaged form each run (N=3) of patient samples using the18s primer set. PD analysis shows all patient samples were significantly different from the NTC (****p<0.0001).

**Table S1**. Nucleotide Sequences for LAMP Primers Targeting 28srRNA (Bio = biotin)

| Primer | Sequences (5’-3’) |
| --- | --- |
| B3 | AACACAAACCCCACGATT |
| F3 | GAGATCCCATAAAAGGTGTTG |
| BIP | CGCTAAAGCGAATTACCGATACCCTACTCATAAAAAGATCTGAGGTA |
| FIP | GGTGAGTTGTTACACACTCCTAAGGTTCATAATGACAGTAGGACG |
| Loop B | AGGCCATAAAAAGGTAGG |
| Loop F | Bio-ATTTCGACTTCCATGACC |

**Table S2.** Nucleotide Sequences for LAMP Primers Targeting 18srRNA (Bio = biotin)

| Primer | Sequences (5’-3’) |
| --- | --- |
| B3 | AGTCGGCATAGTTTATGGT |
| F3 | GCTTAGTTACGATTAATAGGAGTA |
| BIP | GCGAAAGCATTTGCCTAATCTATTTAAGATTACGACGGTATCTGATC |
| FIP | AGTAGTCCGTCTCCAGAAAATCTTACTTTGGGGGCATTCGTATT |
| Loop B | GTTAAGGGAGTGAAGACG |
| Loop F | Bio-TCACCTCTGACATCTG |

**Table S3.** LAMP Master Mix Used for Amplification of Malaria DNA

| Reagent | Concentration |
| --- | --- |
| Sample | 2.5 µL |
| Isothermal amplification buffer II (10X) | 1X |
| Betaine (5M) | 400 mM |
| dNTPs (100 mM) | 2 mM |
| F3 primer (100 µM) | 0.2 µM |
| B3 primer (100 µM) | 0.2 µM |
| FIP primer (100 µM) | 2 µM |
| BIP primer (100 µM) | 2 µM |
| LF primer (100 µM) | 2 µM |
| LB primer (100 µM) | 2 µM |
| EvaGreen (20X) | 0.2X |
| ROX reference Dye (1/10 =50X) | 1X |
| Bst Polymerase 3.0 (8U/ul) | 8 U |
| Nuclease-free H2O | Fill to 25 µL |

**Table S4.** Oligonucleotide Sequences and qPCR Conditions for *var*ATS Assays

|  |  |
| --- | --- |
| Oligonucleotide sequences | **varATS** |
| Primer-For (5’-3’) | CCC ATA CAC AAC CAA YTG GA |
| Primer-Rev (5’-3’) | TTC GCA CAT ATC TCT ATG TCT ATC |
| qPCR reaction conditions | **Concentration** |
| PowerUp™ SYBR™ Green Master Mix | **1x** |
| Primer, 5 µM (each For & Rev) | **Concentration 0.37µM** |
| DNA | **2 µL** |
| Total volume 20 µL | **Total volume 20 µL** |
| qPCR cycling conditions | **Time/Temperature** |
| Pre-incubation | 2 min – 50°C |
| Initial denaturation | 2 min – 95°C |
| Denaturation | 15 sec – 95 °C |
| Annealing | 15 sec – 56°C |
| Elongation | 1 min – 72°C |
| Number of cycles | 40 |
| Melt Curve | 95-60°C, 1.6°C decrement,  60-95°C, 0.05°C increment |

**Table S5.** Asymptomatic Individual Sample Groups and Amplification Results

| Study ID | Group | Microscopy (parasites/µL) | nPCR  (^d^WB) | qPCR (parasite/ µL, WB) | PD-LAMP Significance (N=4) |
| --- | --- | --- | --- | --- | --- |
| 7 | ^a^CC | 0 | 0 | 0 | ns |
| 8 | CC | 0 | 0 | 1 | * |
| 9 | CC | 0 | 0 | 0 | ns |
| 10 | CC | 0 | 1 | 0 | ns |
| 11 | CC | 0 | 1 | 0 | ns |
| 12 | CC | 0 | 1 | 1 | ns |
| 13 | CC | 0 | 1 | 56 | ** |
| 14 | CC | 0 | 1 | 35 | * |
| 15 | CC | 84 | 1 | 12,749 | *** |
| 16 | CC | 612 | 1 | 8.741 | *** |
| 17 | CC | 43,376 | 1 | 100,669 | ** |
| 18 | ^b^CM | 134,902 | not done | 377,406 | * |
| 19 | ^c^SMA | 12,458 | not done | 136,926 | *** |

^a^Community Control; ^b^ Cerebral Malaria; ^c^Severe Malaria Anemia; ^d^Whole Blood

nPCR: 0 = negative for *P. falciparum*, 1 = positive for *P. falciparum*

**Figure S7**. Representative Images of 400 nm streptavidin-coated particles in a LAMP sample imaged on (A) the smartphone device and (B) at 40x objective on an inverted epifluorescence microscope.


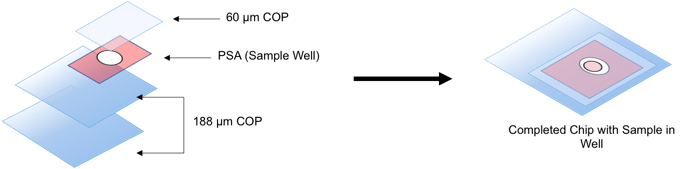


**Figure S8.** Chip Schematic. Layers of 188 µm COP are heat pressed together and hole punched. Double sided PSA is used to form a well for the sample. After sample addition, 60 µm COP adhered to the top of the chip and the sample is imaged in the device.


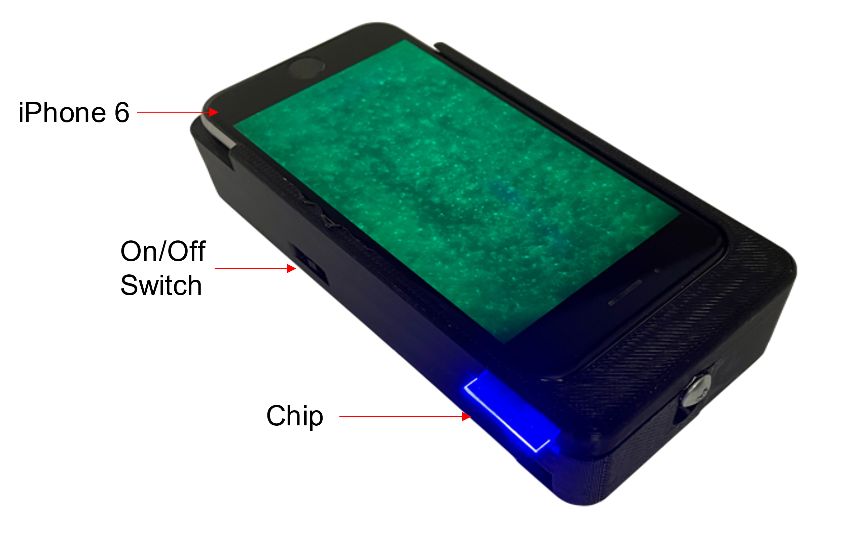


**Figure S9.** Image of Smartphone device and chip
